# On the Relationship Between Attention Processing and P300-Based Brain Computer Interface Control in Amyotrophic Lateral Sclerosis

**DOI:** 10.3389/fnhum.2018.00165

**Published:** 2018-05-28

**Authors:** Angela Riccio, Francesca Schettini, Luca Simione, Alessia Pizzimenti, Maurizio Inghilleri, Marta Olivetti-Belardinelli, Donatella Mattia, Febo Cincotti

**Affiliations:** ^1^Neuroelectrical Imaging and BCI Laboratory, NeiLab, Fondazione Santa Lucia (IRCCS), Rome, Italy; ^2^Servizio Ausilioteca per Riabilitazione Assistita con Tecnologia (SARA-t), Fondazione Santa Lucia (IRCCS), Rome, Italy; ^3^Institute of Cognitive Sciences and Technologies, Consiglio Nazionale delle Ricerche (CNR), Rome, Italy; ^4^Crossing Dialogues, Rome, Italy; ^5^Department of Neurology and Psychiatry, Sapienza University of Rome, Rome, Italy; ^6^Centro Interuniversitario di Ricerca sull’Elaborazione Cognitiva in Sistemi Naturali e Artificiali (ECoNA), Rome, Italy; ^7^ECONA Interuniversity Centre for Reseach on Natural and Artificial Systems, Sapienza University of Rome, Rome, Italy; ^8^Department of Computer, Control and Management Engineering Antonio Ruberti, Sapienza University of Rome, Rome, Italy

**Keywords:** brain-computer interface, amyotrophic lateral sclerosis, attention, event-related potentials, P300, BCI, ALS, EEG

## Abstract

Our objective was to investigate the capacity to control a P3-based brain-computer interface (BCI) device for communication and its related (temporal) attention processing in a sample of amyotrophic lateral sclerosis (ALS) patients with respect to healthy subjects. The ultimate goal was to corroborate the role of cognitive mechanisms in event-related potential (ERP)-based BCI control in ALS patients. Furthermore, the possible differences in such attentional mechanisms between the two groups were investigated in order to unveil possible alterations associated with the ALS condition. Thirteen ALS patients and 13 healthy volunteers matched for age and years of education underwent a P3-speller BCI task and a rapid serial visual presentation (RSVP) task. The RSVP task was performed by participants in order to screen their temporal pattern of attentional resource allocation, namely: (i) the temporal attentional filtering capacity (scored as T1%); and (ii) the capability to adequately update the attentive filter in the temporal dynamics of the attentional selection (scored as T2%). For the P3-speller BCI task, the online accuracy and information transfer rate (ITR) were obtained. Centroid Latency and Mean Amplitude of N200 and P300 were also obtained. No significant differences emerged between ALS patients and Controls with regards to online accuracy (*p* = 0.13). Differently, the performance in controlling the P3-speller expressed as ITR values (calculated offline) were compromised in ALS patients (*p* < 0.05), with a delay in the latency of P3 when processing BCI stimuli as compared with Control group (*p* < 0.01). Furthermore, the temporal aspect of attentional filtering which was related to BCI control (*r* = 0.51; *p* < 0.05) and to the P3 wave amplitude (*r* = 0.63; *p* < 0.05) was also altered in ALS patients (*p* = 0.01). These findings ground the knowledge required to develop sensible classes of BCI specifically designed by taking into account the influence of the cognitive characteristics of the possible candidates in need of a BCI system for communication.

## Introduction

The non-invasive brain-computer interface (BCI) based on the visual event-related potential (ERP) known as P300 (P3; Farwell and Donchin, 1988) is by far the most extensively investigated BCI system to enhance or even allow communication when this latter is severely compromised due to different neurological disorders (Kleih et al., 2011; Riccio et al., 2016). Despite the large amount of studies seeking for methods to ultimately optimize P3-based BCI control accuracy, a reliable and yet flexible (to be customized for individual users) BCI system still requires some research efforts.

Within the range of users in need of a BCI for communication and control, those with amyotrophic lateral sclerosis (ALS) represent *the target* population due to a progressive muscular paralysis that leads to a loss of communication and interaction ability thus preventing persons from using conventional assistive technologies (ATs) at the later stage of the disease.

A number of studies reported that ALS patients can communicate by using a P3-based BCI (Marchetti et al., 2013) with stable performance over time (Sellers and Donchin, 2006; Nijboer et al., 2008; Silvoni et al., 2013). Communication and interaction could also be enhanced by means of a P3-based BCI combined with an AT device (Thompson et al., 2014; Schettini et al., 2015). Marchetti and Priftis (2015) reported the results of a meta-analysis (pooled studies from 2008 to 2013) indicating that the effectiveness of the P3-speller (Farwell and Donchin, 1988) in ALS patients reached an overall classification accuracy of 73.72%. Further studies aimed at investigating predictors of P3-based BCI control in ALS patients showed that both external (the stimuli exploited) and internal factors (the user’s motivation) could account for the BCI performance (Nijboer et al., 2010; Townsend et al., 2010; Kaufmann et al., 2013b). Conversely, the relation between BCI performance and the clinical-functional status of ALS patients (Nijboer et al., 2008; Silvoni et al., 2013; McCane et al., 2014; Thompson et al., 2014) has not been fully investigated yet. McCane et al. (2015) reported no significant differences in BCI accuracy between ALS patients and healthy age- (but not years of education-) matched subjects. However, they found differences in the target-related ERPs characteristics: the ALS group presented a higher N2 wave peak amplitude and a latency delay in N2, P3 and late negativity (LN) with respect to the control group. However, studies comparing ALS patients with healthy participants are still scarce. Further investigations are needed on the possible impairment/alteration of brain processing in response to external inputs (such as visual stimuli) delivered within a BCI framework of stimulation to eventually unveil whether and how they could influence the BCI control.

In this regard, it is important to note that cognitive deficits have been described in ALS patients (Lomen-Hoerth et al., 2003; Ringholz et al., 2005; Christidi et al., 2012; Strutt et al., 2012; Volpato et al., 2016; Radakovic et al., 2017). Up to now, the influence of ALS patients’ cognitive profile on the visual P3-based BCI control has not been fully investigated. The current studies on visual P3-based BCIs for communication often involved end-users with severe motor disabilities due to neurological disorders of various etiology (Piccione et al., 2006; Zickler et al., 2011; Kaufmann et al., 2013b; Kübler et al., 2014; Riccio et al., 2015) as compared to those studies in which more homogenous groups of participants, such as only ALS patients, were enrolled (Sellers and Donchin, 2006; Nijboer et al., 2008; Riccio et al., 2013; Silvoni et al., 2013; Thompson et al., 2014; Schettini et al., 2015). As such, this inconsistency between studies does not allow for definitive inferences on how P3-based BCI control could vary in severely motor disabled end-users with ALS and how this variability could be related to cognitive processing. In this line of reasoning, we previously showed (Riccio et al., 2013) how some aspects of attention processing such as the stimulus *temporal filtering* (i.e., the ability to keep the attentional filter active during the selection of a target) would be a predictor of the P3-speller control accuracy in ALS patients. Since we did not include a control (healthy) group, we could only speculate that such temporal aspect of attention processing was impaired in ALS population by comparing our results with those reported in other studies which included healthy participants (Kranczioch et al., 2005; Georgiou-Karistianis et al., 2007).

In the present study, we investigated whether the accuracy in mastering a P3-based BCI by an ALS population sample would be affected by the previously identified alterations in the attention processing and whether these alterations would be exclusive of the ALS population. To this purpose, we compared a group of ALS patients with a group of healthy volunteers both controlling a P3-speller (Farwell and Donchin, 1988). Groups were matched for age and years of education since both factors are known to deeply influence performance in executing cognitive tasks (Ardila et al., 2000). As yet, the two groups underwent an identical BCI stimulation protocol (i.e., the sequence of stimuli were not customized) in order to avoid confounding factors due to the use of different stimulation protocols. Based on previous findings (Riccio et al., 2013), our present hypothesis was that the ALS patients would show an altered visual attention processing of the stimuli delivered during the P3-based BCI control, and this would, in turn, affect the ability to control the P3-based BCI (i.e., decrease in performance). We also investigated the possible relation between cognitive mechanisms and P3-speller control to further corroborate the role of cognitive dysfunctions in BCI control in ALS patients (Riccio et al., 2013).

## Materials and Methods

### Participants and Baseline Assessment

Thirteen participants (8 males; mean age 62.2 ± 13; years of formal education 13.7 ± 5.1) with ALS diagnosis (ALS group) and 13 age and years of education-matched participants (9 males; mean age 55.3 ± 9; years of formal education 13.3 ± 3) with no history of neurological/psychiatric disorders (Control group) were enrolled in the study. Seven out of 13 ALS patients participated in the previous study (Riccio et al., 2013).

The ALS patients were recruited through the ALS Center of the Policlinico “Umberto I”, Sapienza University, Rome. The study was conducted at Fondazione Santa Lucia, IRCCS, Rome and approved by the Independent Ethics Committee of Fondazione Santa Lucia. All participants (or the legal representatives of ALS patients when required) provided a written informed consent.

The inclusion criterion for the ALS patients was the ability (also with the help of an AT device if required) to clearly communicate (at least) a binary response (yes/no). Patients with other concomitant neurological or psychiatric disorders, any impediment in the acquisition of electroencephalography (EEG) data from the scalp (e.g., wounds, dermatitis), severe concomitant pathologies (fever, infections, metabolic disorders, severe heart failure), or episodes of reflex epilepsy were excluded from the study.

The level of physical disability was assessed by means of the “ALS Functional Rating Scale-Revised” (ALSFRS-R; Cedarbaum et al., 1999). ALSFRS-R scores range from 0 to 48 (the higher the score, the higher the functionality). Mean ALSFRS-R score was 31.2 ± 10.4 (range from 12 to 41). The ALS patients’ demographic and clinical information are reported in Table 1.

**Table 1:** Participants’ characteristics.

	ALS group	Control group
Age (years)	62.2 ± 13 (40–80)	55.3 ± 9 (44–68)
Sex (M/F)	8/5	9/4
Years of formal education	13.7 ± 5.1 (5–18)	13.4 ± 3.4 (8–18)
EF (impaired/not impaired)	5/6	5/8
EF (perseverative responses)	88.5 ± 16.5	96.6 ± 20.2
EF (total errors)	88.5 ± 14.3	93.6 ± 15.4
SA (impaired/not impaired)	3/7	1/12
SA (errors)	2.4 ± 2.9	0.8 ± 1.9
WM (impaired/not impaired)	4/5	1/10
WM (omissions)	3.8 ± 3.8	1.6 ± 2.0
ALSFRS-R	31.2 ± 10.4 (12–41)	-
Onset (S/B)	5/8	-
Time since diagnosis (mo)	26.8 ± 22.6	-

*Demographic and clinical characteristics of the ALS group and Control group (means ± standard deviations, range). Abbreviations: F, female; M, male; SA, selective attention; EF, executive functions; WM, working memory; S, spinal; B, bulbar*.

Participants underwent a cognitive assessment focused on attention domains, in order to have individual baseline profiles. Two clinical neuropsychological tests were applied for the cognitive screening. The computerized test for attentional performance (TAP; Zimmermann and Fimm, 1995) was used to assess selective attention (SA) and working memory (WM) whereas the executive functions (EF) were assessed by means of the perseverative response scores obtained in the Wisconsin Card Sorting Test (WCST; Berg, 1948). Between the several clinical tests, the TAP includes a go-nogo task for SA (participants had to press a key when two target items were presented, while ignoring three distracter items) and a 2-back task for WM (numbers were presented on the screen and participants had to indicate the repetition of a number within an interval of three numbers by pressing a key). The WCST test consists of a card sorting game according to either color, shape or number. The sorting rule changes over time. Participants then have to rely on the outcome or feedback after each of their choice in order to infer the new rule in effect. Eight of the 13 ALS patients completed the full protocol (psychological session and BCI session); the remaining five performed only the BCI session (one patient participated to the earlier study—Riccio et al., 2013). All Control subjects (*n* = 13) had both the psychological session and the BCI session.

### Experimental Session

The experimental design consisted of two separate sessions (performed on two different days): the BCI session and the psychological session (see below for details).

### BCI Session

Scalp potentials were acquired by means of a 16-channel amplifier (g.MOBILAB, g.tec, Austria) from eight active electrodes (g.Ladybird, g.tec, Austria) placed according to 10–10 international standard (Fz, Cz, Pz, Oz, P3, P4, PO7, and PO8; right ear lobe reference, left mastoid ground). This experimental choice was dictated by a reasonable trade-off between a not exhausting experimental procedure for ALS patients and a widely accepted eight electrodes configuration to ensure a P300 based-BCI successful control. Signals were digitized at 256 Hz. Stimulus paradigm and online delivery were managed by means of the BCI2000 framework (Schalk et al., 2004). A P3-speller (Farwell and Donchin, 1988) interface (6 by 6 matrix of alphanumeric items) was displayed full screen on a 15 computer screen, placed approximately at eye level and at a distance of 100 cm from the participant.

During the calibration phase (i.e., no feedback on performance), the subjects had to focus on 15 items forming three predefined words (3 runs; 5 items for each run). The target to focus on was shown to the participants by a single flash, after which rows and columns were randomly intensified for 125 ms, with an inter stimulus interval (ISI) of 125 ms. Participants were suggested to mentally count how many times that target was flashing. Calibration data were segmented into epochs lasting 800 ms (time 0 marked the stimulus onset) that were fed into a stepwise linear discriminant analysis (SWLDA) to determine the classifier coefficients (Krusienski et al., 2006) to be applied in the online BCI session. During the online phase (i.e., provision of feedback on performance), participants had to spell four predefined (copy mode) words (4 runs; 5 items for each run; 20 characters in total). The classifier coefficients extracted from calibration data were applied to epochs grouped by stimulation classes (rows and columns) and averaged over stimulation sequences. The spelled letter was identified as the intersection between the row and the column exhibiting the maximum of the sum of scored features (Krusienski et al., 2006). As mentioned in the Introduction section, the stimulation sequences were not customized for each patient and thus we performed a static data collection (Mainsah et al., 2015). Specifically, a single item (e.g., letter) was intensified 20 times (10 sequences) before the next set of stimuli would start. Such stimulation sequence was maintained fixed for each subject.

### Psychological Session

The individual temporal pattern of attentional resource allocation was tested by means of a rapid serial visual presentation (RSVP) paradigm which corresponds to an attentional blink (AB) paradigm in Kranczioch et al. (2007). In brief, it consisted of two target stimuli (T1 and T2) which were embedded in a stream of 16 or 19 distracter stimuli; each stream of stimuli (the equivalent of a single trial) was presented in the center of a monitor (white background). All stimuli were capital letters (letters F, K, Q, X, Z were excluded) and were presented pseudo-randomly (with a constraint that the same letter was not presented within three sequential positions) at central fixation (1 stimulus/100 ms; presentation rate at 10 Hz). As for target stimuli, T1 was a green capital letter randomly occurring as 4th, 5th, 6th or 7th item within a single stream. T2 was a black capital “X” which followed T1 on 80% of the trials according to four conditions (each occurring with a frequency of 20%): after no intervening distracters, after one, three or five intervening distracters. In 20% of the trials, T2 was not presented (5th condition). The distracter stimuli were black capital consonants.

Upon the stimulus stream delivery, participants were asked to answer the following questions: (1) whether the green letter (T1) was a vowel (T1 was a vowel on 50% of the trials); and (2) whether the black X (T2) was contained in the stimulus stream. In the case of ALS patients, the answers to the questions were given according to their residual motor activity (e.g., verbal response, head movements, eye movements).

Twenty practice trials preceded a total of 160 experimental trials (32 trials for each of the five T2 conditions); these latter were fully randomized within two presentation blocks separated by a pause of 5 min.

## Data Analysis

All acquired data were preprocessed as follows. High and low pass filters (4th order Butterworth filter) were applied with a cut off frequency of 1 Hz and 20 Hz, respectively. EEG signals with peak amplitude higher than 70 μV or lower than −70 μV were removed. Data were then segmented into epochs (time 0 denoted the stimulus onset) lasting 800 ms and 1000 ms for the BCI and ERPs analysis, respectively. Both target and non-target stimulus-related epochs were considered.

### BCI Performance Analysis

The BCI online accuracy was expressed as the percentage of correct selections (i.e., the ratio between the number of correct selections and the total number of selections). Furthermore, an offline estimation of both the accuracy and the information transfer rate (ITR, Wolpaw et al., 2002) was performed in order to account for the online performance inter-subject variability that was “hidden” by the static modality of data collection.

To estimate the offline accuracy, a baseline correction was performed based on the mean amplitude of signal within the 200 ms pre-stimulus interval. The offline accuracy was then calculated for each stimulation sequence by means of a 7-fold cross-validation technique according to which six runs were used as training dataset to extract SWLDA classifier parameters and one run was used as testing dataset. A mean accuracy value for each stimulation sequence was obtained by averaging the values resulting from the seven iterations.

The ITR (bits/min) was estimated for each subject and each stimulation sequence based on the definition of bit-rate as in Wolpaw et al. (2002) and multiplying the bit-rate by the speed of selection (selections/minute). The individual highest ITR value was considered. Output metric resulting from this computation will be reported as ITR (0–800 ms).

The relative contribution of the N2 and P3 ERP components to the BCI accuracy was investigated offline as follows. Differences in the amplitudes of ERPs that were elicited by the stimulus types (target vs. non-target) were quantified using the coefficient of determination R2. We considered the epochs relative to all seven runs. The R2 values range from 0 to 1, wherein higher values correspond to larger explained variances. A *signed* R2 index was introduced to account for the different polarity of ERPs (N2 and P3) and was derived by multiplying R2 by the sign of the slope of the corresponding linear model which was positive when the amplitudes of the ERPs that were elicited by the target stimuli were higher than those elicited by non-target stimuli and vice versa (as in Aloise et al., 2012). The mean *R*^2^ values were computed within two different temporal intervals for the N2 and P3 components that ranged from 100 ms to 400 ms for the N2 (negative values) and from 250 ms to 550 ms for the P3 (positive values).

As for the ITR, its values were computed by segmenting data into epochs between 0 ms and 550 ms after the stimulus onset—ITR (0–550 ms)—to ensure that the temporal interval would include both N2 and P3 ERP components.

### ERPs Analysis

We focused the ERP analysis on the N2 as the earliest ERP that reliably correlates with visual awareness (Visual Awareness Negativity; Railo et al., 2011) and the P3 as associated with conscious access to the content of conscious vision (Raffone et al., 2014). Hence, these two ERP components can be considered a reliable reflection of attentional stimulus processing.

In this offline ERP analysis, the epochs in which a target stimulus occurred within the 500 ms preceding the stimulus onset were removed in order to reduce the contamination between consecutive epochs and the ERP overlapping (Treder and Blankertz, 2010). Target and non-target ERP waveforms were obtained by averaging the epochs relative to each run. The ERP waveforms were obtained from a sample by sample contrast between the non-target and target ERP waveform amplitude (i.e., the difference between target and non-target).

The mean of both the N2 and P3 amplitude and the centroid latency (Luck, 2005) were obtained from all data sets (seven BCI session runs). The P3 mean amplitude was calculated by averaging the voltage of all positive points preceded or succeeded by positive values between 250 ms and 550 ms after the stimulus onset whereas for the N2 mean amplitude we averaged the voltage of all negative points preceded or succeeded by negative values between 100 ms and 400 ms after the stimulus onset (Clayson et al., 2013). The P3 and N2 centroid latencies were set as the time to which the area under the curves was divided into equal halves. Finally, the mean amplitude and the centroid latency of the P3 waves (P3-MA and P3-CL, respectively) recorded from Fz, Cz and Pz and the Mean Amplitude and the Centroid Latency of the N2 waves (N2-MA and N2-CL, respectively) recorded from PO7, PO8 and Oz were subjected to the statistical analysis.

### Psychological Paradigm Data Analysis

As for the RSVP data set, the accuracy of T1 and T2 detection (T1%; T2%) was estimated (T2% was considered only in trials in which T1 had been correctly identified). T1% was considered an index of participants’ temporal attentional filtering capacity and T2% was considered as an index of the capability to adequately update the attentive filter (Riccio et al., 2013) in the temporal dynamics of the attentional selection.

### Statistical Analysis

Between-group (ALS and Control) differences in terms of BCI control performance were evaluated as follows. A (non-parametric) Mann-Whitney U test was applied to assess the between-group difference in BCI online accuracy (accuracy scores not normally distributed). A Student’s *T*-test was applied to assess the between-group difference in terms of the ITR scores. The contribution of N2-R2 and P3-R2 to the BCI performance was assessed for each group (ASL and Control) by means of two linear regression analyses with the ITR (0–550 ms) as the dependent variable and the N2-R2 and the P3-R2 as independent variables.

The (non-parametric) Spearman’s rank order correlation was applied to investigate the possible correlation between ALSFRS-R scores (not normally distributed) and the ITR values.

To investigate whether ALS patients showed differences with respect to Control group in attention processing during the BCI task, we conducted two MANOVAs to determine the effect of group (independent variable) on both P3-MA and P3-CL (dependent variables). The same analysis (two MANOVAs) was performed to determine the effect of group on both N2-MA and N2-CL.

To investigate whether the temporal pattern of attentional resource allocation would be correlated with the BCI performance level (ITR), we performed a correlation analysis (Pearson’s correlation coefficients) between the ITR (0–800 ms), the P3-MA in Pz and T1% and T2%. Such correlation was sought either by pooling all data from ALS and Control groups and by considering only ALS group data.

The existence of alterations in attentional resources allocation in the ALS group was assessed by means of a MANOVA to test the effect of group (independent variable) on T1% and T2% (dependent variables).

## Results

The ALS and Control groups did not show significant differences as regard demographic characteristics (Student’s *T*-test; *t*_(24)_ = 1.6; *p* = 0.13 and *t*_(24)_ = 0.17; *p* = 0.85 for age and “years of formal education”, respectively) and clinical assessment focused on selective attention (SA; error scores, *χ*^2^ = 1.96; *p* = 0.16), working memory (WM; errors *χ*^2^ = 3.29; *p* = 0.07) and executive functions (EF; *χ*^2^ = 0.119; *p* = 0.729).

**Figure 1:**
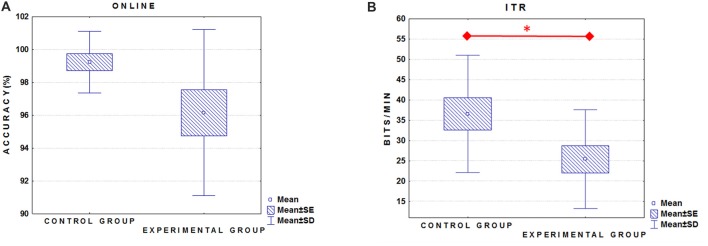
Box plots illustrate the comparison between groups relative to the online performance **(A)** and information transfer rate (ITR) **(B)**. Experimental group = amyotrophic lateral sclerosis (ALS) patients. *Indicates significant difference (*p* < 0.05).

We did not find a significant between-group difference in the online accuracy (Figure 1A; ALS group mean = 96.1% ± 5; Control group mean = 99.2% ± 2; *U* = 55.5; *p* = 0.13). On the contrary, ITR (0–800 ms) was significantly higher in the Control group (36.6 ± 14.5 bits/min) as compared to ALS group (mean = 25.4 ± 12.1 bits/min; *t*_(24)_ = 2.1, *p* ≤ 0.05; Figure 1B).

**Figure 2:**
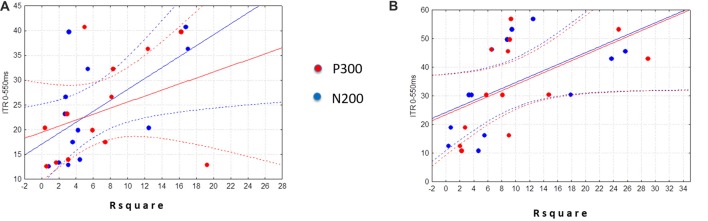
Scatter plots illustrate the relationship of the ITR (0–550 ms) with the N2Rsquare (blue dots) and the P3Rsquare (red dots) in the ALS (experimental group) **(A)** and in the Control group **(B)**. Note that regression analysis was performed by considering only the absolute values of R2 (i.e., no *signed* R2 was considered).

The linear regression analysis (*p* = 0.11) revealed that ITR (0–550) was not significantly predicted neither by the N2-R2 values (*β* = 0.33; *p* = 0.34) nor the P3-R2 values (*β* = 0.32; *p* = 0.34) in the Control group (Figure 2B). Differently, in the ALS group we found that the linear regression was significant (*F*_(2,10)_ = 4.3526, *p* < 0.05). Specifically, only the N2-R2 was significantly predictive of the ITR (0–550 ms; *β* = 0.59, *p* < 0.05; P3-R2 = *β* = 0.34, *p* = 0.17; Figure 2A).

We found that the ITR and ALSFRS-R scores showed a high tendency to correlate which did not reach a significance (*p* = 0.06; *r* = 0.52). Such tendency, however, suggests that the degree of disability due to ALS could influence the BCI performance in a detrimental way.

No significant differences were found between ALS and Control groups (MANOVAs) in P3-MA (*λ* = 0.71; *F*_(3,22)_ = 2.9, *p* = 0.05), N2-MA (*λ* = 0.88; *F*_(3,22)_ = 0.9; *p* = 0.4) and N2-CL (*λ* = 0.89; *F*_(3,22)_ = 1.0; *p* = 0.4). On the contrary, the MANOVA returned a significant between-group difference in P3-CL (*λ* = 0.59; *F*_(3,22)_ = 5.0; *p* < 0.01) values over Fz (*p* < 0.001; Control group mean = 363.17 ± 20.14 ms; ALS group mean = 409.4 ± 38.07 ms; Bonferroni corrected) and Cz electrodes (*p* < 0.05; Control group mean = 368.60 ± 27.62 ms; ALS group mean = 396.68 ± 32.66 ms; Bonferroni corrected) with longer CL in ALS patients with respect to Controls. No significant differences were found in P3-CL values over Pz (*p* = 0.3; control group mean = 379.71 ± 37.83 ms; ALS group mean = 394.4 ± 38.82 ms; Bonferroni corrected).

**Figure 3:**
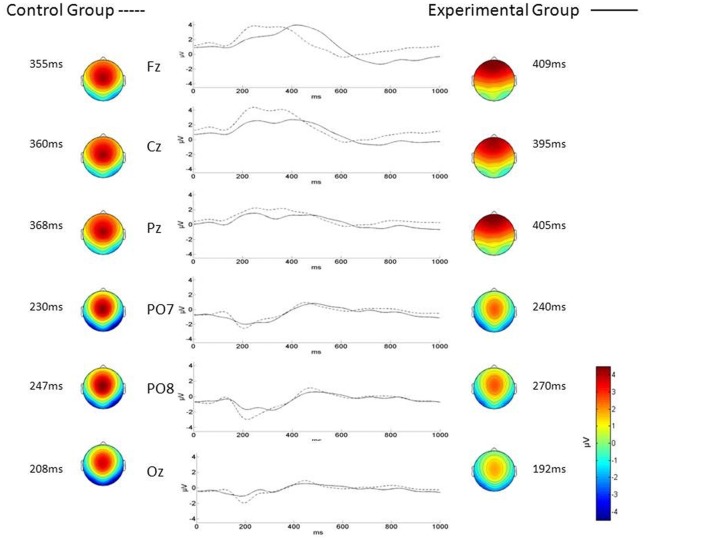
P300 topography and waveforms in ALS (Experimental Group) and Control group. Traces in the middle panel represent the grand average of the difference between target and non-target electroencephalography (EEG) amplitude as a function of time (interval between 0 = stimulus onset and 1000 ms) recorded for ALS (*n* = 13 patients; solid line) and Control group (*n* = 13 subjects; dotted line) during the brain-computer interface (BCI) session, over different electrode positions (left labels). The maps represent the scalp topographical distribution in Control (left) and ALS (right) group of the P3 and N2 centroid latency (grand average) over Fz, Cz and Pz and over PO7, PO8 and Oz respectively.

As illustrated in Figure 3, the visual inspection of P3 topography indicates a prevalent frontal distribution of P3 in ALS whereas a parietal distribution is observed in Control group.

The analysis of the relation between cognitive substrates and BCI performance as measured by means of RSVP and BCI data returned a significant positive correlation between T1% and the ITR (*r* = 0.51; *p* < 0.05) and T1% the P3-MA in Pz (*r* = 0.63; *p* < 0.05) when considering all group data (ALS and Control data pooled). No significant correlation was found between T2% and ITR (*r* = 0.32; *p* = 0.15) and between T2% and P3-MA (*r* = 0.23; *p* = 0.31). When considering only the ALS group, the same analysis unveiled significant positive correlation between T1% and ITR (*r* = 0.71; *p* < 0.05) and T1% and P3-MA (*r* = 0.78; *r* < 0.05) whereas T2% and ITR (*r* = 0.66; *p* = 0.07) and T2% and P3-MA (*r* = 0.39; *p* = 0.31) did not show significant correlation.

The MANOVA (*F* = 4.4; *p* < 0.05) revealed that the T1% values were significantly lower in ALS as compared to Control group (Control group mean 89.7 ± 0.8%; ALS group mean = 79.4 ± 10%; *p* = 0.01; Bonferroni corrected). No significant difference was found in T2% (control group mean = 63.6 ± 2%; ALS group mean = 67.8 ± 23%; *p* = 0.6).

### Discussion

This study aimed at investigating whether ALS patients showed differences in the ability to control a P3-speller BCI system with respect to healthy subjects. We focused on the attention processing involved in the delivering of the visual BCI stimulation paradigm, in order to further (Riccio et al., 2013) elucidate if and how such cognitive abilities would be altered in ALS patients and eventually would account for patients’ BCI control capacity. We hypothesized that the capacity to accomplish a P3-speller task was decreased in ALS patients and that they would have shown an alteration in the visual attention processing as elicited during the P3-based BCI control. To test our hypothesis, we compared two groups of participants (ALS patients vs. Control) in terms of performance in P3-speller control and with regard to the earliest ERP components such as N2 and P3 which are correlated with visual awareness (Railo et al., 2011; Raffone et al., 2014).

First, we found that the ALS patients showed a significantly lower ITR in the P3-speller BCI task with respect to Controls whereas the online performance was comparable between the two groups.

This finding is not in line with what reported by McCane et al. (2015). According to their study severely disabled ALS patients and age-matched healthy controls showed similar P3-based BCI performance in terms of maximum accuracy, communication rate and bit rate. Several differences between these two studies might account for the apparent discrepancy on the ability to master a P3-based BCI by ALS patients. First, the pattern of visual stimulation (checkerboard in McCane et al., 2015) that is well known to remarkably influence the P3-based BCI control performance (Townsend et al., 2010) and related ERPs characteristics (Kaufmann et al., 2011). Second, the ALS clinical severity that was higher in ALS population of McCane (ALSFRS-R scores = 9.4 ± 9.5 SD) with respect to our population (ALSFRS-R scores = 31.2 ± 10.4). In this regard, we found a remarkable (but not significant) correlation between the ITR and the ALSFRS-R scores that suggests a direct relation between the degree of clinical disability and the ability to use a P3-based BCI. Finally, in McCane et al. (2015) study, the ALS and healthy participants were not matched for years of formal education and this is a variable also accounting for the level of cognitive task performance (Ardila et al., 2000).

In addition to this, the overall methods (and metrics) to estimate the P3-based BCI performance are not directly comparable between the two studies. We “only” found the (offline) ITR as a distinctive metric of the ability to use a P3-based BCI in ALS with respect to Control group.

In the P3-speller task, the act of focusing attention on the target letter modulates the visual processing of the stimulus. Our ERP findings indicate that the P3 mean latency was significantly higher in ALS with respect to control group while no difference was found in the N2 parameters between the two groups. The finding of a delayed P3 associated with a “normal” N2 (i.e., physiological stimulus categorization process) in ALS can be interpreted as a *delay* occurring in the post-perceptual stage of the stimulus attentional processing (Duncan-Johnson and Kopell, 1981) that is, the variation in the attention modulation during the stimulus visual processing observed in ALS would occur when stimulus perception is complete, the target is categorized and its storing in WM has taken place. We found no significant between-group differences in P3 mean amplitude; this latter parameter can be considered as a measure of the attention allocation resources (Donchin, 1981). This finding allows us to speculate that in ALS patients the overall alteration of attention modulation during a P3-speller task is only related to the time of processing but not to the resource allocation.

The P3 wave component showed a frontal topography in the ALS group as compared to the parietal distribution observed in the Control group (Figure 3). This finding is in line with previous findings reported by McCane et al. (2015). We interpret this difference in P3 topography as possibly related to the P3a and P3b components that have different generators and thus, different topography (Courchesne et al., 1975): the *frontal* P3 component observed in the ALS group would represent the P3a whereas the *parietal* P3 present in Controls would better represent the P3b component.

Fabiani and Friedman (1995) suggested that during the process of learning a task, the P3a is elicited by novel stimuli that do not require a memory template. In contrast, the P3b is elicited when the stimulus memory template is created. One can speculate that the *frontal* P3a topography in ALS might reflect an increase in the frontal activity related to a more rapid decay of the memory templates (of the stimuli processed) that would make it more difficult to create and maintain an adequate template for the target stimulus (Fabiani and Friedman, 1995; Fabiani et al., 1998).

We found that the N2-R2 and not the P3-R2 coefficient significantly predicted the BCI accuracy only in ALS group, accounting for the 59% of the ITR variance. Based on the assumption that such coefficients mostly returned the contribution of N2 and of P3 waves to the BCI classification performance, these findings indicate that in ALS patients the N2 elicited during the P3-speller task would have a major role with respect to P3 in successful target selection.

The presence of a jitter in the P3 latency has been described in healthy subjects controlling a P3-based BCI system and its magnitude would be correlated with the online performance (Thompson et al., 2013; Aricò et al., 2014). Although it remains to be demonstrated that such P3 wave jitter exists and to what extent it might influence the P3-speller BCI task performance in ALS patients, one can speculate that our observed *unbalance* in the contribution of N2 and P3 components to successful BCI performance in favor of N2 might reside in the cross-relationship between the P3 latency jitter and delayed visual processing phenomena. Hence, further investigations in this regard are of utmost relevance to address sensible design of future ERP-based BCIs for ALS user candidates.

According to our previous findings (Riccio et al., 2013), the temporal aspect of the SA investigated by means of a RSVP task and measured as T1% (i.e., the ability to maintain the attentional filter active during the selection of a target within a range of time) was found to be related to both the BCI performance and P3 amplitude in ALS patients.

In the present study, we confirmed that the temporal filter in attention processing of visual stimuli in ALS patients was altered, by directly compare the T1% and T2% values obtained from ALS and Control group. Specifically, we found that the capability to detect T1 (but not T2) was lower in the ALS group.

As such, this finding is consistent with that of a delay in the P3 latency which reflects a deficit in the temporal aspect (i.e., post-perceptual stage of the stimulus attentional processing) of the context update. Taken altogether, these findings clearly indicate the existence of an alteration in the temporal aspects of the visual stimulus processing as presented in a “conventional” P3-speller matrix in ALS population and that this time-related alteration in the capacity to temporally process visual stimuli does influence the rate of success in BCI control.

Our findings might lay the groundwork for future clarification of some of the relevant issues in the actual deployment of ERPs-based BCI for communication to ALS users, such as the impact of end-users’ cognitive profile in designing user-centered (Liberati et al., 2015; Nijboer, 2015) and reliable BCI systems (Kübler et al., 2013).

### Study Limitation

Some limitations pertaining different aspects of this study deserve to be mentioned. First, our ALS population does not include ALS patients in a complete locked-in state (LIS). Although this restriction in the inclusion criterion was mandatory to allow the cognitive screening, it prevents any generalization of our findings to those patients with no means of communication (i.e., complete LIS). In this framework, our study suggests a possible role of the cognitive assessment to be performed in ALS patients before they would be in a LIS condition, including the specific cognitive abilities identified here as critical for P3-based BCI usage.

Second, our findings relative to BCI factors influencing BCI performance allow us to make inferences only regarding the control of a P3-speller in a group of ALS patients and cannot be generalized to the control of other BCIs. It is conceivable that when exploiting different features to control other BCIs, temporal aspects of attention would not have a comparable role. An example of “alternative” features to P3 would be the N400 wave, involved in the elaboration of meaningful stimuli such as face recognition (Kaufmann et al., 2013b). In addition, several single-case studies have shown significant differences in classification performance depending on the sensory modality of the ERP-based BCIs that participants controlled (Kaufmann et al., 2013a; Schreuder et al., 2013).

Third, attention is a complex domain of the cognitive functions (Posner, 1975) and its substrates can be measured in different ways; this prevents from a direct comparison of results obtained within different contexts and with different behavioral and neurophysiological approaches. For instance, attention substrates measured with different tests (i.e., Cognitrone, Schuhfried, 2007) were not found to be precursors of performance in controlling sensorimotor–based BCIs, differently from visuo-motor coordination (Hammer et al., 2012, 2014).

## Conclusion

This study involved a group of participants with ALS and a group of healthy participants matched for age and years of formal education. Our results showed that both the capacity to accomplish the P3-speller task and the timing of the allocation of attentional resources in the post-perceptual stage of stimulus processing were altered in ALS patients. Furthermore, we confirmed that the capacity to temporally filter a target stimulus within a stream of stimuli was related to a lower capacity for ALS to control a P3-speller.

Developing AT devices that restore communication in people with severe motor disabilities is a central issue of BCI research (Millán et al., 2010). Current BCI systems do not address the heterogeneity of the end-users often due to the lack of customizability and adaptability to their cognitive capabilities. This study contributes to the knowledge needed for developing a new class of BCI specifically designed by taking into account the influence of the cognitive characteristics of end-users on BCI usage.

## Author Contributions

AR was responsible for experimental design, data collection, analysis of data and manuscript writing. FS was responsible for data acquisition and analysis. LS was responsible for behavioral assessment (attention tasks). AP and MI were responsible for the patients’ enrolment. MO-B, DM and FC supervised the overall experimental design implementation, data interpretation and manuscript editing.

## Conflict of Interest Statement

The authors declare that the research was conducted in the absence of any commercial or financial relationships that could be construed as a potential conflict of interest.
